# Identification of core genes and transcription factors related to metabolic reprogramming in atherosclerosis: a multi-omics analysis and experimental validation approach

**DOI:** 10.3389/fmolb.2026.1756851

**Published:** 2026-02-25

**Authors:** Yanhong Liu, Yirong Ma, Zhijian Song, Junyu Lai, Yingying Huang, Doukun Ding, Zengguang Fan, Jianguang Wu

**Affiliations:** 1 Department of Postgraduate, Jiangxi University of Chinese Medicine, Nanchang, China; 2 Cardiology Department, Affiliated Hospital of Jiangxi University of Chinese Medicine, Nanchang, China

**Keywords:** Atherosclerosis, bioinformatics, machine learning algorithms, metabolic reprogramming, multi-omics analysis

## Abstract

**Background:**

Atherosclerosis (AS) is a chronic inflammatory disease driven significantly by metabolic reprogramming (MR). However, the core MR-related genes and their specific functions in AS remain incompletely understood, thus creating an urgent need for reliable diagnostic and therapeutic biomarkers.

**Methods:**

Two AS-related microarray datasets (GSE100927 and GSE28829) were integrated and normalized. Differential expression analysis identified differentially expressed genes (DEGs), which were intersected with an MR-related gene set to obtain MR-related DEGs (MRDEGs). Functional enrichment analyses—including Gene Ontology (GO) and Kyoto Encyclopedia of Genes and Genomes (KEGG) analyses—were conducted. Subsequently, weighted gene co-expression network analysis (WGCNA) was combined with multiple machine learning algorithms to screen for hub genes. These candidate genes were further validated using an external dataset (GSE43292) and evaluated via receiver operating characteristic (ROC) curve analysis. Additionally, a multi-gene diagnostic model was constructed and assessed using both nomogram and SHAP analysis. Single-gene Gene Set Enrichment Analysis (GSEA) elucidated the biological functions of core genes. Immune infiltration and single-cell analyses investigated microenvironment remodeling. Moreover, transcription factor (TF) prediction via hTFtarget, integrated with transcriptome sequencing of human umbilical vein endothelial cells (HUVECs), identify upstream regulators. Finally, Experimental validation was performed in ApoE^−/−^ mice.

**Results:**

We identified 57 MRDEGs and selected four core genes—LYN, FABP5, MMP9, and ANPEP—which demonstrated high diagnostic value. The multi-gene model showed strong clinical predictive performance. GSEA further revealed significant involvement of these genes in immune-inflammatory pathways. Immune infiltration and single-cell analyses confirmed substantial immune microenvironment remodeling and altered cell-cell communication. EGR1 was identified as a key upstream transcription factor. Ultimately, Experimental validation in ApoE^−/−^ mice confirmed marked upregulation of all four core genes at mRNA and protein levels, with EGR1 also showing significantly elevated protein expression.

**Conclusion:**

This study identifies LYN, FABP5, MMP9, and ANPEP as core MR-related genes in AS, clarifies their roles in immune microenvironment regulation, and confirms their value as diagnostic biomarkers, thereby providing new insights for precise diagnosis and targeted therapy of AS.

## 1 Introduction

Atherosclerosis (AS) is a chronic inflammatory disease of the arterial intima. It is typically manifested by lipid deposition, proliferation of smooth muscle cells (SMCs), and fibrous tissue hyperplasia, ultimately leading to the formation of arterial plaques, luminal narrowing, and impaired blood flow (Jebari-Benslaiman et al., 2022). As a common pathological basis for various cardiovascular diseases, AS can directly contribute to severe conditions such as hypertension, myocardial infarction, and ischemic stroke (Virani et al., 2020). According to global epidemiological statistics, cardiovascular diseases account for up to 18 million deaths annually, consistently ranking as the leading cause of human mortality (Di Cesare et al., 2024). Notably, alterations in lifestyle and diet, factors including elevated consumption of sugar, fat, protein, and cholesterol, alongside growing smoking and alcohol use, have thereby contributed to the rising global incidence of AS, with a noticeable trend toward earlier onset (Chen W. et al., 2023). Current clinical management of AS primarily involves pharmacological interventions and surgical treatments. Drug therapy regimens include lipid-lowering agents (such as statins, ezetimibe, and PCSK9 inhibitors), antiplatelet agents, microcirculation-improving drugs, and thrombolytics (Libby, 2021). However, even with standardized lipid-lowering therapy, the risk of major cardiovascular and cerebrovascular events is reduced by only approximately 30%, and a considerable proportion of patients still experience plaque progression. Antiplatelet therapy increases the risk of gastrointestinal bleeding, while interventional procedures such as angioplasty, stent implantation, and bypass grafting, although improving perfusion, are limited by complications including postoperative restenosis and thrombosis (Crea, 2023; Borovac et al., 2019; Ma et al., 2023). Hence, gaining a comprehensive insight into the pathogenesis of AS and establishing dependable prognostic indicators for vulnerable populations are paramount for enabling early intervention, timely diagnosis, and decelerating the progression of the disease.

The pathogenesis of AS involves multiple molecular and cellular mechanisms, including lipid infiltration and modification, inflammatory responses, oxidative stress, and cell death. Among these, metabolic dysregulation is not only a central feature but may also drive disease progression through interactions with other mechanisms. Under physiological conditions, cardiac energy metabolism primarily relies on mitochondrial oxidative phosphorylation of fatty acids, while other metabolites such as glucose, amino acids, and ketone bodies also contribute to energy supply. However, under pathological conditions, significant reprogramming of metabolic pathways and preferences occurs, manifesting as metabolic remodeling or reprogramming (Gandoy-Fieiras et al., 2020). The development and advancement of AS are intimately linked to metabolic reprogramming (MR) in macrophages, SMCs, and endothelial cells (Dai et al., 2024). Within the pathological microenvironment, infiltrated macrophages exhibit enhanced glycolysis to meet energy demands, accompanied by impaired mitochondrial oxidative phosphorylation. This metabolic shift not only promotes the release of pro-inflammatory cytokines (e.g., IL-1β, TNF-α) but also increases the generation of reactive oxygen species (ROS), which further oxidize low-density lipoprotein (LDL) to form more pathogenic oxidized LDL (oxLDL). Excessive oxLDL uptake causes defective cholesterol efflux in macrophages, promoting cholesterol ester deposition and irreversible foam cell formation—a key event in lipid core development and inflammation amplification (Vendrov et al., 2024; Bekkering et al., 2014). SMCs shift from oxidative phosphorylation to glycolytic dependency, supporting their proliferation, migration, and extracellular matrix synthesis. Some SMCs even transdifferentiate into macrophage-like phenotypes and similarly uptake lipids to form foam cells, further exacerbating intraplaque lipid accumulation and structural complexity (Bennett et al., 2016). Metabolic disturbances and oxidative stress in endothelial cells compromise nitric oxide bioavailability, leading to endothelial dysfunction. This dysfunction manifests as diminished vasodilation, heightened platelet aggregation, increased monocyte adhesion and infiltration, along with lipoprotein accumulation beneath the intima. These events collectively initiate early atherosclerotic lesions (Zhao et al., 2024). Notably, lipid raft microdomains on the cell membrane serve as signaling platforms and play a key role in integrating metabolic stress and inflammatory signals, thereby regulating endothelial NO synthesis and thrombotic propensity. Studies have shown that specific pathological stimuli (such as autoantibodies) can inhibit endothelial nitric oxide synthase (eNOS) phosphorylation via a lipid raft-dependent LRP8 receptor signaling pathway, exacerbating NO synthesis impairment and pro-thrombotic phenotypes (Riitano et al., 2023). These intertwined MR processes create a vicious cycle of “metabolic dysfunction–inflammatory response–lipid deposition–cellular impairment,” ultimately driving plaque growth, lipid core expansion, and increased instability. Given its central role in disease progression, systematically elucidating the key regulatory genes and networks involved in MR may facilitate the identification of reliable diagnostic biomarkers and inspire novel interventional strategies.

**FIGURE 1:**
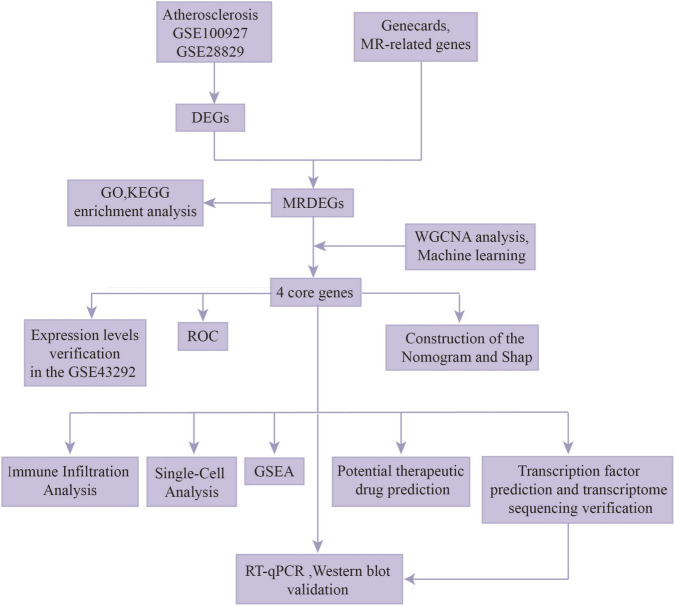
Workflow of the analysis.

Nevertheless, although the critical role of MR in AS has been recognized, several key issues remain unresolved. First, the core gene regulatory network driving MR in AS has not been systematically elucidated. Second, most existing studies have focused on single pathways or cell types, lacking an analytical framework centered on MR that integrates multi-omics data to reveal its global regulatory mechanisms. Finally, whether molecular changes associated with MR can be translated into robust diagnostic biomarkers with clinical utility—specifically for early screening, patient risk stratification, or plaque vulnerability assessment—remains to be explored. Based on these challenges, we propose the central hypothesis that the progression of AS is governed by a core MR module composed of key genes and their upstream transcription factors. This module serves as a molecular hub linking immune–metabolic dysregulation with plaque progression and holds potential as an effective diagnostic biomarker. To test this hypothesis, we developed a multi-omics integrative analytical framework with MR as the focal point. By integrating transcriptomic, single-cell sequencing, and immune microenvironment data, we aimed to systematically map the overall landscape of metabolic regulatory networks in AS. Methodologically, we established a screening pipeline that combines weighted gene co-expression network analysis (WGCNA) with multiple machine-learning algorithms to enhance the robustness of key biomarker discovery. Using this approach, we not only identified a core gene signature consisting of *LYN* proto-oncogene, Src family tyrosine kinase (*LYN)*, fatty acid binding protein 5 (*FABP5)*, matrix metallopeptidase 9 (*MMP9)*, and alanyl aminopeptidase, membrane (*ANPEP)* but also revealed the transcription factor early growth response 1 (*EGR1*) as a potential common upstream regulatory node for these genes, providing new insights into the transcriptional cascade governing MR. A diagnostic model constructed based on these four core genes suggested good discriminative performance in an independent validation cohort, offering potential experimental support for the development of novel molecular tools for early AS screening and risk stratification (Figure 1). In summary, this study adopted a research paradigm that integrates multi-omics data, computational screening, and experimental validation to systematically uncover key regulatory genes and networks involved in AS-MR, thereby providing novel insights for a deeper understanding of its pathological mechanisms and the development of innovative diagnostic strategies.

## 2 Materials and methods

### 2.1 Data acquisition and process

The GEO database (www.ncbi.nlm.nih.gov/geo/) provided the microarray datasets of AS patients, including the GSE100927, GSE28829, and GSE43292 datasets. The GSE100927 dataset, derived from human peripheral arteries, consists of 69 AS samples and 35 healthy arterial samples. The GSE28829 dataset originates from human carotid arteries, comprising 16 advanced atherosclerotic plaque samples and 13 early plaque samples. The GSE43292 dataset, also from human carotid arteries, includes 32 atherosclerotic plaque samples and 32 control samples (Steenman et al., 2018; Doring et al., 2012; Ayari and Bricca, 2013).

Batch effect correction was performed on the combined GSE100927 and GSE28829 datasets utilizing the “SVA” package in R software, ensuring the removal of non-biological variations (Chakraborty et al., 2012). An independent validation set, GSE43292, was employed. Genes implicated in MR were selected from the GeneCards database based on a keyword search for “metabolic reprogramming” and a relevance score greater than 4, resulting in a final set of 227 genes (Supplementary Table S1).

### 2.2 Analysis of differential expression

The limma package was used to identify the differentially expressed genes (DEGs) between the AS and control groups. The screening criteria were |logFC| > 1 and an adjusted P-value <0.05. To create MR-related DEGs (MRDEGs), the resultant DEGs were then intersected with MR-related genes. Gene Ontology (GO) and Kyoto Encyclopedia of Genes and Genomes (KEGG) enrichment studies were carried out using the “ClusterProfiler” package in order to better clarify the biological relevance of the MRDEGs.

### 2.3 Weighted gene Co-expression network analysis (WGCNA)

A systems biology method called WGCNA is used to examine gene expression data by building co-expression networks and locating gene modules linked to particular characteristics or illnesses. Using the “WGCNA” package in R, we conducted WGCNA in this study to assess physiologically significant co-expression networks among DEGs (Langfelder and Horvath, 2008). The network type was set to “unsigned,” the minimum module size was set to 60, the merge cut height was set to 0.25, the deepSplit parameter was set to 2, and the optimal soft-thresholding power was set to 10.

### 2.4 Machine learning-based feature selection

Three machine learning techniques were used in this investigation to assess and rank diagnostically relevant hub genes: RF (implemented using the “randomForest” R package), LASSO regression (using the “glmnet” R package), and SVM-RFE (using the “e1071″R package). In particular, SVM-RFE used 10-fold cross-validation for recursive feature reduction,RF chose features based on the mean decrease in Gini impurity, and LASSO regression selected features by adjusting the lambda value. All three approaches found overlapping genes, which were kept as potential hub genes.

### 2.5 Validation using an external dataset

We used the external dataset GSE43292 to confirm the important genes that were discovered. We then used the “pROC” R program to analyze the receiver operating characteristic (ROC) curve and assess the degree of correlation between AS pathological state and gene expression patterns. The degree of relationship was measured using the area under the curve (AUC) value.

### 2.6 Analysis of single-gene enrichment

KEGG-derived gene sets were used to conduct single-gene enrichment analysis on the identified important genes. Using the “clusterProfiler” package in R, which determined the association p-value for each gene with respect to the gene sets, enrichment analysis was carried out via the hypergeometric distribution. Gene sets were deemed substantially enriched if their adjusted p-value was less than 0.05.

### 2.7 Analysis of immune infiltration

To describe the distribution of different immune cell subtypes among samples, we used the CIBERSORT algorithm to deconvolute and estimate the relative abundance of immune cell subtypes within the combined dataset (Kawada et al., 2021). To further investigate the relationship between immune cell infiltration and sample characteristics, the Kruskal–Wallis test was applied to assess the significance of differences in immune cell abundance among different groups. Subsequently, to assess expression relationships between various immune cell types, correlation analysis was carried out using R’s “cor” function.

### 2.8 Single-cell analysis

The dataset GSE155512 was derived from atherosclerotic carotid artery tissues obtained from three patients who underwent carotid endarterectomy (Pan et al., 2020). The R package “Seurat” (version 4.4.3) was used for data processing (Butler et al., 2018). Tight quality control standards were used to guarantee the quality of the data: cells with mitochondrial UMI counts more than 15% and those with less than 500 or more than 8,000 genes were not included. The data were then log-normalized using default parameters. To find the top 2000 most variable genes, the “FindVariableGenes” tool was then used. The “ElbowPlot” function was used to calculate the number of principle components, and the “RunPCA” function was used to perform principal component analysis (PCA). The top 20 principal components (pcSelect = 20) were chosen for further examination. For visualization, t-SNE plots were generated using the “RunTSNE” function, and heatmaps were created based on gene expression within each cluster. For additional analysis, the core genes' expression patterns were also mapped onto the t-SNE projections. Finally, cell-cell communication analysis was conducted using CellCha to explore signaling networks among different cell populations (Jin et al., 2021). CellChat constructs intercellular communication networks based on a known ligand–receptor interaction database and computes communication probabilities between distinct cell types.

### 2.9 Small molecule drug regulatory network analysis and TF prediction

In this work, we screened for possible therapeutic drugs that target the discovered biomarkers using the DGIdb database (http://www.dgidb.org) (Freshour et al., 2021), thereby identifying novel therapeutic targets. The resulting drug-target interactions were visualized using Cytoscape (version 3.10.1). We used the hTFtarget platform (http://bioinfo.life.hust.edu.cn/hTFtarget#!/), which offers a comprehensive resource for discovering TF–gene interactions based on experimentally supported and projected data, to suggest TFs that might be regulating the core genes. Each of the four core genes was individually analyzed for TF binding predictions. Through intersection analysis, we further identified TFs with potential regulatory effects on all four core genes (Supplementary Material 2).

### 2.10 Cell preparation

Human umbilical vein endothelial cells (HUVECs) that had been cryopreserved were quickly thawed in a water bath at 37 °C, moved to a biosafety cabinet, and then carefully pipetted to suspend the cells in the cryovial. After that, the cell suspension was moved to a 15 mL centrifuge tube, combined with three times the amount of the freezing solution of new complete medium, and centrifuged for 5 minutes at 250 × g. After being aspirated, the supernatant was thrown away. The cell pellet was moved to a culture flask for incubation after being reconstituted in new DMEM media enhanced with 10% fetal bovine serum (FBS). Following cell attachment, the cells underwent two PBS washes, and the supernatant was disposed of. To break down the cells until the cell borders started to separate, trypsin was applied. The digestion was then stopped by adding fresh complete medium. After carefully pipetting the cells to ensure complete separation, they were moved to a 15 mL centrifuge tube and centrifuged for 5 minutes at 250 × g. The cells were resuspended in new complete media and subcultured at a 1:3 ratio after the supernatant was disposed of. During the logarithmic growth phase, HUVECs were collected after being digested with trypsin and cleaned with PBS. For 5 minutes, the cell suspension was centrifuged at 250 *g*. To create a single-cell suspension, the pellet was resuspended in a suitable volume of full medium. Cells were cultivated overnight at 37 °C with 5% CO_2_ after being seeded at the proper densities into 96-well or 6-well plates. ECM endothelial-specific media was used in place of the original medium following cell attachment. The model group received treatment with basal media enriched with 150 μM oxLDL to mimic AS, while the blank control group received simply 10% FBS-containing basal medium.

### 2.11 Transcriptome sequencing analysis

Before building a library, total RNA was taken from each experimental group’s HUVECs and put through a quality check. Initially, the RNA was broken up into roughly 300 bp segments. Following the sequential synthesis of first-strand and second-strand cDNA, PCR amplification and size selection of library fragments (∼450 bp) were performed to finalize library preparation. The Agilent 2,100 Bioanalyzer was used to evaluate the libraries' quality and concentration. Using a paired-end 150 bp (PE150) method, qualified libraries were sequenced on the Illumina NovaSeq platform, producing over 15 Gb of raw data per sample. Platform-specific software was used to transform the raw sequencing data to FASTQ format. High-quality clean readings were obtained by performing quality control using Fastp to exclude adaptor contamination and low-quality sequences (Q < 20). Using the proper aligner, these clean reads were matched to the human reference genome (GRCh38, Ensembl v108.38). Fragments Per Kilobase of transcript per Million mapped reads, or FPKM, was used to quantify and standardize the levels of gene expression.

### 2.12 Animal modeling and grouping

Experimental animals were provided by the Sibeifu (Beijing) Biotechnology Co., Ltd. Laboratory Animal Center (License No.: SCXK (Jing) 2024-0001). The study employed twenty male mice who were 8 weeks old. There were ten C57BL/6J mice kept on a regular diet in the blank control group. The AS model was established by feeding 10 ApoE^−/−^ mice a high-fat diet for 12 weeks. All animals were kept under barrier conditions with unrestricted access to food and water (temperature: 22 °C ± 2 °C, humidity: 50%–60%, 12-h light/dark cycle, specified pathogen-free [SPF] facility). The Ethics Committee of Jiangxi University of Chinese Medicine gave its approval to the experimental procedures (Approval No.: 20240312025).

### 2.13 RT-qPCR and total RNA extraction

Following the guidelines provided by the RNA extraction kit’s manufacturer, total RNA was extracted from cell or tissue samples. A Nanodrop 2000 spectrophotometer was used to measure the RNA’s concentration and purity. SweScript All-in-One RT SuperMix for qPCR (with gDNA Remover; Servicebio, Wuhan, China) was used to reverse-transcribe equal amounts of RNA from each sample into cDNA. The 2× Universal Blue SYBR Green qPCR Master Mix (Servicebio, Wuhan, China) was used to conduct quantitative real-time PCR on a Bio-Rad CFX Connect Real-Time PCR System. Pre-denaturation at 95 °C for 30 s, 40 cycles of denaturation at 95 °C for 15 s, and annealing/extension at 60 °C for 30 s comprised the thermal cycling conditions. A melting curve analysis was subsequently conducted to validate amplification specificity. Every reaction was carried out in triplicate. GAPDH was used as the internal reference gene in the ΔΔCt technique to determine the relative gene expression levels. The Supplementary Material 3 contains primer sequences.

### 2.14 Western blot (WB)

The protein expression levels of Lyn, Fabp5, Mmp9, Anpep, Egr1, and the internal control β-actin were ascertained by WB analysis. In short, RIPA lysis buffer including protease and phosphatase inhibitors was used to extract the total proteins from cells or tissues, and the BCA Protein Assay Kit was used to measure the protein concentrations. On 10% SDS-PAGE gels, equal quantities of protein were separated and then transferred to PVDF membranes. The membranes were blocked for 1 hour using 5% non-fat milk, and then they were treated with primary antibodies for the whole night at 4 °C. The membranes were cleaned and then incubated for 1 hour at room temperature with goat anti-rabbit or goat anti-mouse secondary antibodies conjugated with HRP. An ECL chemiluminescence detection reagent was used to see the protein bands, and ImageJ software was used to quantify the band intensities.

### 2.15 Statistical analysis

R software was used to organize and analyze the data. The two independent samples t-test was used to evaluate statistical differences between the AS group and the control group. The following criteria were used to determine statistical significance: *p < 0.05, **p < 0.01, and ***p < 0.001.

## 3 Results

### 3.1 Identify DEGs related to MR

**FIGURE 2:**
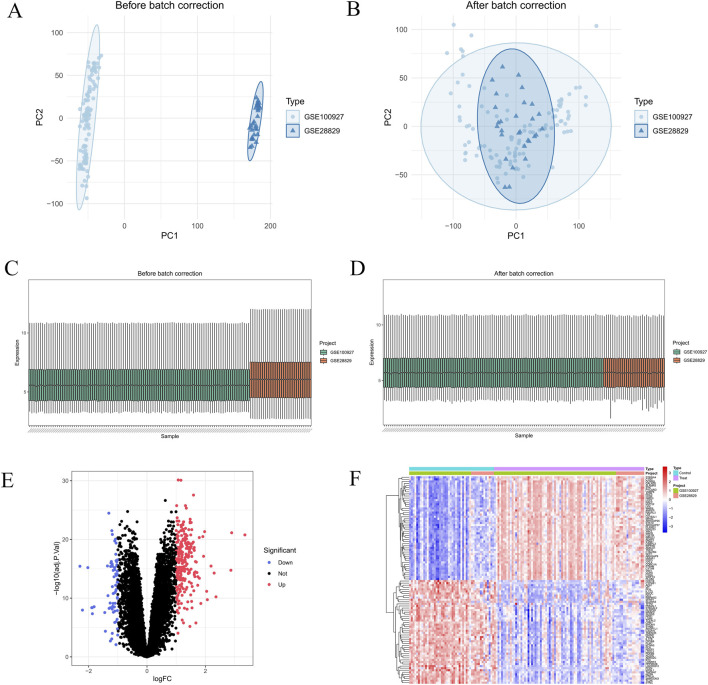
Identification of DEGs. **(A,B)** Two-dimensional PCA cluster plot of GSE100927 and GSE28829 datasets before and after normalization. **(C,D)** Boxplot of GSE100927 and GSE28829 datasets before and after batch correction. **(E)** Volcano plot of DEGs. Red spots represent upregulated genes and blue spots represent downregulated genes. **(F)** Heatmap of DEPs and DEGs.

**FIGURE 3:**
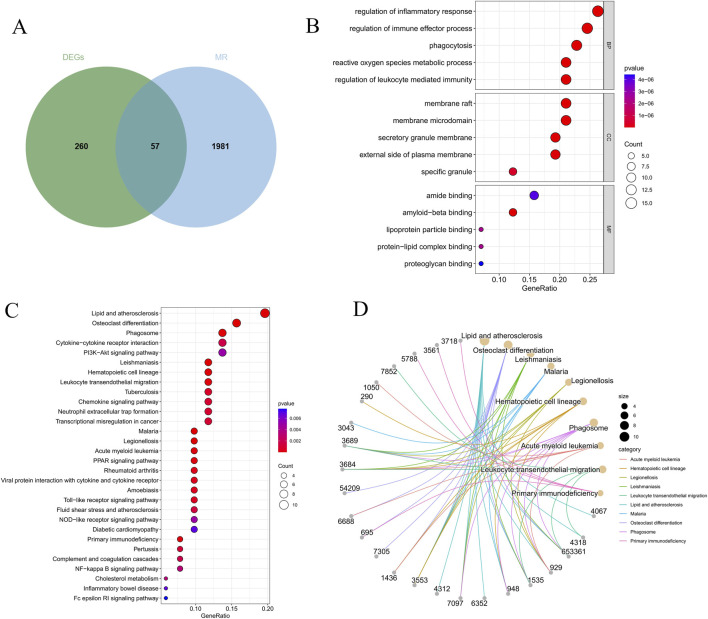
Enrichment analysis and identifying differentially expressed MRDEGs. **(A)** Venn plot of common genes between DEGs and MR-related genes. **(B)** Enriched GO analysis items.”BP”, biological processes; “CC”, cellular component; “MF”, molecular functions. **(C,D)** Enriched items from the KEGG pathway analysis items.

In order to remove batch effects, the GSE100927 and GSE28829 datasets were first combined. The data before and after merging were visually compared using boxplots and principal component analysis (PCA) plots (Figures 2A–D). The limma software was then used to conduct a differential gene expression analysis between the control and AS groups, and the findings were displayed in a volcano plot (Figure 2E). Next, we intersected the AS-related DEGs with a known set of MR-related genes, identifying 57 MRDEGs (Figure 3A). These genes were mostly implicated in biological processes such ROS metabolic control, immunological effector function, and inflammatory response, according to GO enrichment analysis (Figure 3B). Significant enrichment in important immune-inflammatory signaling pathways, such as PI3K–Akt, PPAR, NF–kappa B, and Fc epsilon RI pathways, was found by KEGG pathway analysis, confirming their strong correlation with inflammatory and immunological responses (Figures 3C,D).

### 3.2 Identification of candidate hub genes via WGCNA

**FIGURE 4:**
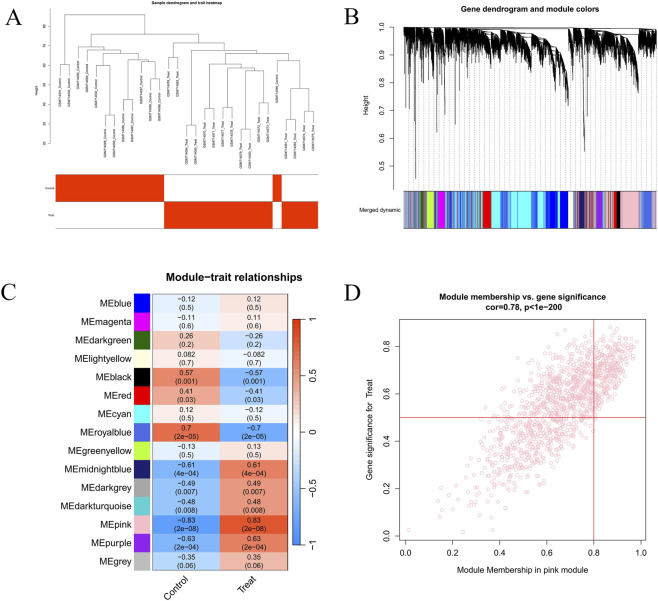
WGCNA. **(A)** Sample clustering dendrogram with trait heatmap showing control and treatment groups. **(B)** Gene dendrogram with module colours representing different co-expression modules. **(C)** Heatmap of correlations between modules and traits. **(D)** Scatter plot of module membership versus gene significance in the pink module.

WGCNA was performed on the GSE28829 dataset. Initially, the top 8,000 genes with the highest median absolute deviation (mad) values were selected, and the optimal soft-thresholding power was set to 10. Using this parameter, 15 distinct gene modules were identified. Based on module–trait correlation analysis (assessed by correlation coefficient r and p-value), the ME pink module was identified as the most significantly associated with AS (r = 0.83, p = 2e-14) (Figures 4A–D). Genes within the ME pink module were subsequently considered key AS-related genes. These were further intersected with the MRDEGs, resulting in the identification of 43 candidate hub genes.

### 3.3 Identification and validation of core genes

**FIGURE 5:**
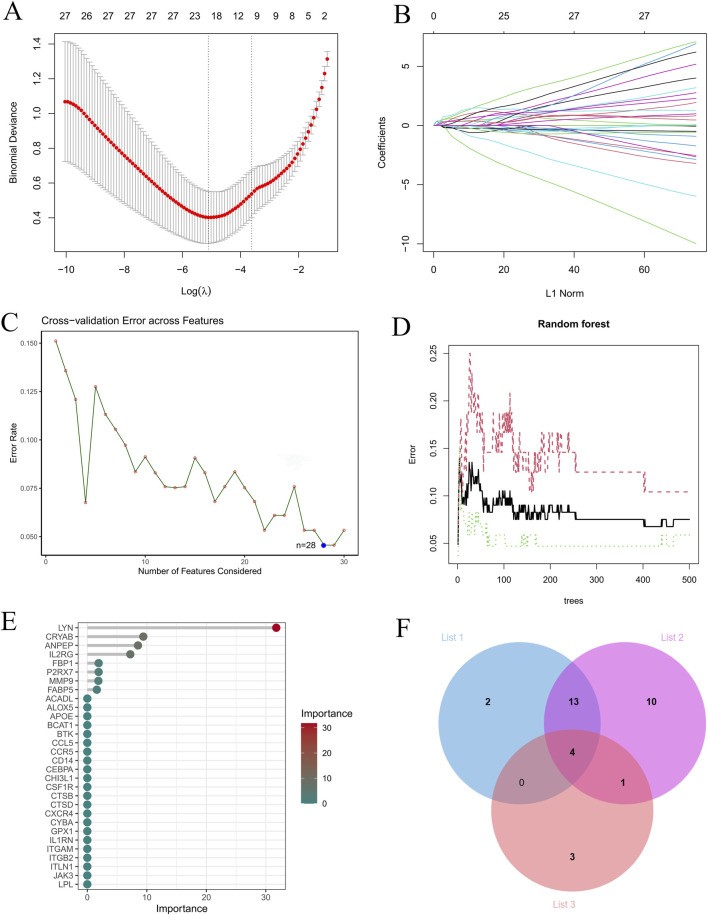
Identify hub genes associated with metabolic reprogramming using machine learning models. **(A)** LASSO regression for feature selection. **(B,C)** SVM-RFE for optimal feature selection. **(D,E)** RF analysis showing error rates and feature importance ranking. **(F)** Venn diagram showing the intersection of genes identified by the three algorithms.

**FIGURE 6:**
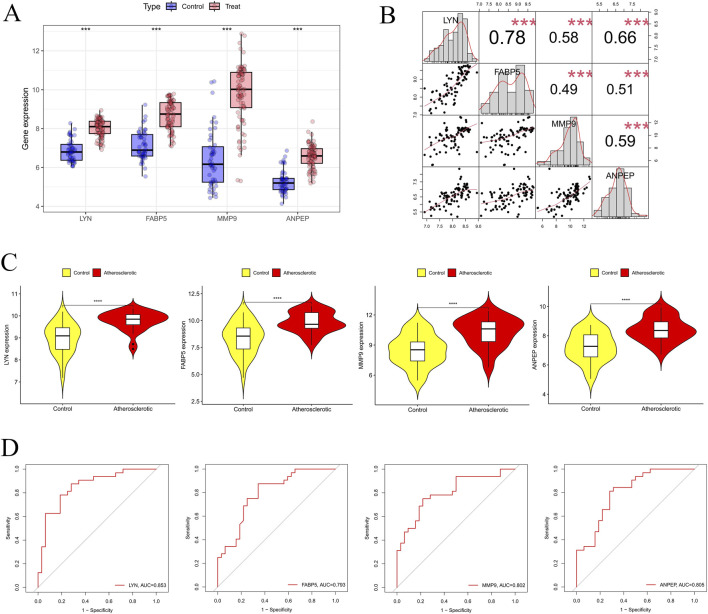
Validation and diagnostic value of hub genes. **(A)** Expression levels of *LYN*, *FABP5*, *MMP9*, and *ANPEP* between control and atherosclerotic groups. **(B)** Correlation analysis among the four hub genes. **(C)** Validation of hub gene expression in independent dataset. **(D)** ROC curves evaluating the diagnostic performance of *LYN*, *FABP5*, *MMP9*, and *ANPEP*. Significance levels are indicated as *P < 0.05; **P < 0.01; ***P < 0.001.

Multiple machine learning algorithms, including LASSO (Figures 5A,B), SVM-RFE (Figure 5C), and RF (Figures 5D,E), were applied to screen for core genes from the 43 candidate genes. The LASSO analysis identified 19 genes, SVM-RFE selected 28 genes, and RF ranked the top eight genes based on importance. By taking the intersection of genes identified by these three methods via Venn analysis, four core genes—*LYN*, *FABP5*, *MMP9*, and *ANPEP*—were ultimately determined (Figure 5F). To visualize their expression patterns, box plots were generated using the merged dataset. The results indicated that all four core genes were significantly upregulated in the AS group compared to the control group (Figure 6A). Interestingly, correlation analysis further revealed strong positive correlations among these core genes (Figure 6B).

To further validate the reliability of these candidate core genes, the external dataset GSE43292 was utilized. Fortunately, the AS group had considerably higher expression of all four genes (p < 0.001) (Figure 6C). Furthermore, their diagnostic performance was assessed using ROC curve analysis. The AUC values were 0.853 for *LYN*, 0.793 for *FABP5*, 0.803 for *MMP9*, and 0.805 for *ANPEP* (Figure 6D). Overall, these core genes exhibit strong diagnostic potential, suggesting their possible value in early identification and risk prediction of AS.

### 3.4 Construction of the nomogram and SHAP

**FIGURE 7:**
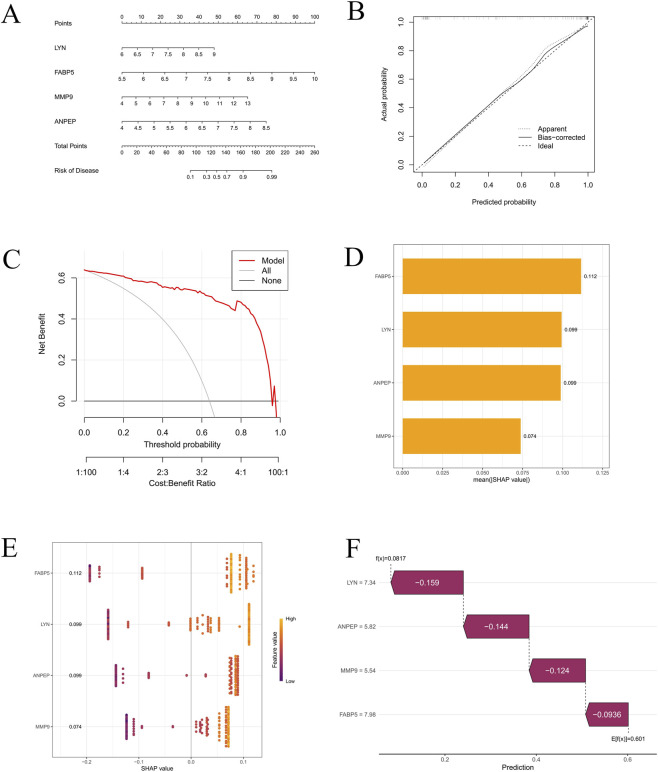
Construction and evaluation of the diagnostic model based on hub genes. **(A)** Nomogram integrating *LYN*, *FABP5*, *MMP9*, and *ANPEP* for predicting disease risk. **(B)** Calibration curve showing good agreement between predicted and actual probabilities. **(C)** DCA demonstrating the clinical net benefit of the model. **(D)** Barplot of SHAP values indicating the relative importance of hub genes. **(E)** Scatter plot of SHAP values showing gene-level contributions. **(F)** SHAP summary plot visualising the impact of each hub gene on model prediction.

A nomogram was constructed using the four identified core genes based on the merged dataset (Figure 7A). Each gene corresponds to a point scale, and the sum of these points is converted into an individual’s risk of developing the disease. A higher total score indicates increased disease risk (Figure 7B). A clinical impact curve (CIC), calibration curve (Figure 5B), and decision curve analysis (DCA) were used to assess the nomogram’s predictive performance. All three showed that the nomogram had a strong predictive potential for coronary artery disease (CAD) (Figure 7C).

To develop an interpretable model with SHAP, we first merged three datasets—GSE100927, GSE28829, and GSE43292—and removed batch effects. Training (70%) and validation (30%) sets were randomly selected from the combined dataset. Ten machine learning models were trained and evaluated, including partial least squares (PLS), RF, support vector machine (SVM), logistic regression, k-nearest neighbors (kNN), XGBoost, neural network, gradient boosting decision tree (GBDT), generalized boosted regression models (GBM), and gradient boosting (gboost). With an AUC of 0.910, the GBM model performed the best among them. The relative importance of each gene in the model’s predictions is illustrated in a SHAP summary bar plot, while a beeswarm plot depicts the distribution of SHAP values versus feature values for each core gene, reflecting the magnitude and direction of their contributions across different values. The results indicated that FABP5 may play the most substantial role in predictive outcomes (Figures 7D,E). Finally, a waterfall plot displays the model’s prediction function for an individual instance (f(x) = 0.0817) along with the baseline expected value (E [f(x)] = 0.601) (Figure 7F).

### 3.5 Single-gene Gene Set Enrichment Analysis (GSEA) enrichment analysis

**FIGURE 8:**
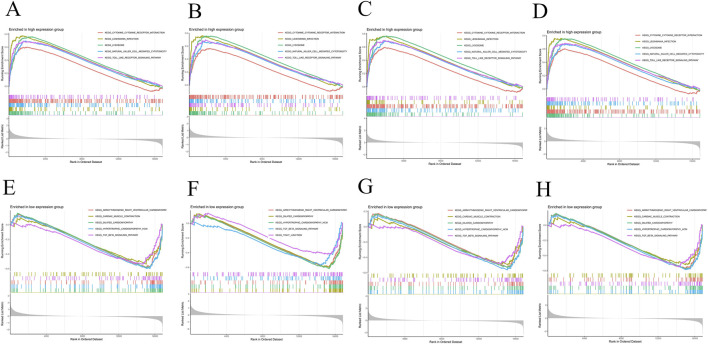
GSEA of hub genes. **(A–D)** Pathways enriched in the high-expression groups of *LYN*, *FABP5*, *MMP9*, and *ANPEP*. **(E–H)** Pathways enriched in the low-expression groups of *LYN*, *FABP5*, *MMP9*, and *ANPEP*.

Each of the four core genes (*LYN*, *FABP5*, *MMP9*, and *ANPEP*) in this study underwent single-gene GSEA. The results revealed that these genes were significantly enriched in multiple key biological pathways, particularly in Cytokine-Cytokine Receptor Interaction, Leishmania Infection, Lysosome, Natural Killer Cell Mediated Cytotoxicity, and Toll-like Receptor Signaling Pathway. These pathways are strongly linked to immunological and inflammatory responses, indicating that by influencing immune and inflammatory processes, *LYN*, *FABP5*, *MMP9*, and *ANPEP* may be crucial for the development, course, and stability of AS. Notably, the natural killer cell-mediated cytotoxicity pathway and the Toll-like receptor signaling pathway may play crucial roles in the immune-inflammatory reactions that underlie AS, offering fresh insights into the molecular involvement of these genes in the illness (Figures 8A–H).

### 3.6 Analysis of immune-related status and genes

**FIGURE 9:**
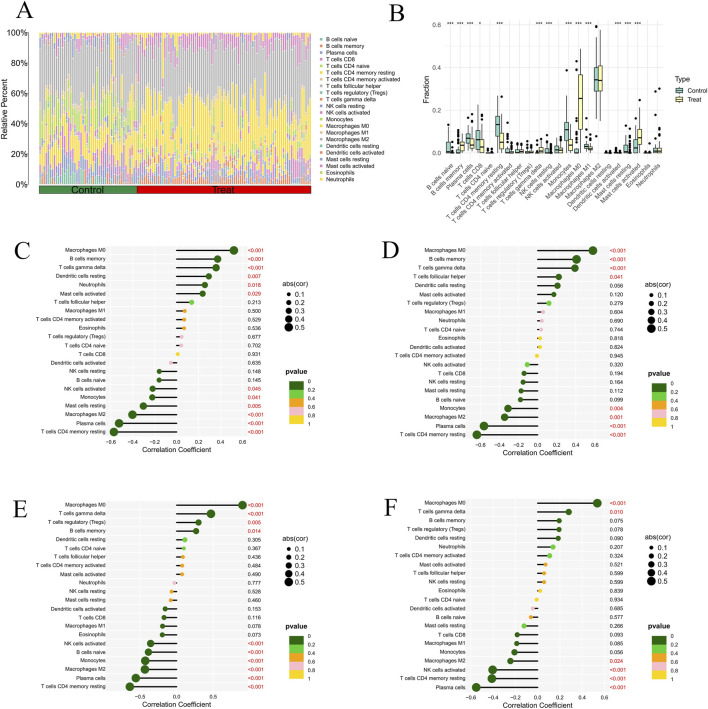
Immune infiltration analysis. **(A)** Stacked barplot showing the relative proportions of 22 immune cell types in control and treatment groups. **(B)** Boxplot comparing immune cell fractions between groups. **(C–F)** Correlations between hub gene expression (*LYN*, *FABP5*, *MMP9*, and *ANPEP*) and infiltrating immune cells.

B cells, T cells, natural killer (NK) cells, macrophages, and mast cells were among the 22 immune cell types whose infiltration levels were significantly different between AS and normal samples (Figures 9A,B). Most of these immune cells exhibited a downward trend in expression within AS samples. In addition, a lollipop plot was created to show the relationships between the invading immune cells and the four key genes. The findings indicated that these four core genes had substantial negative associations with resting CD4 memory T cells, plasma cells, and M2 macrophages, and significant positive correlations with M0 macrophages and γδ T cells (Figures 9C–F).

### 3.7 Single-cell analysis results

**FIGURE 10:**
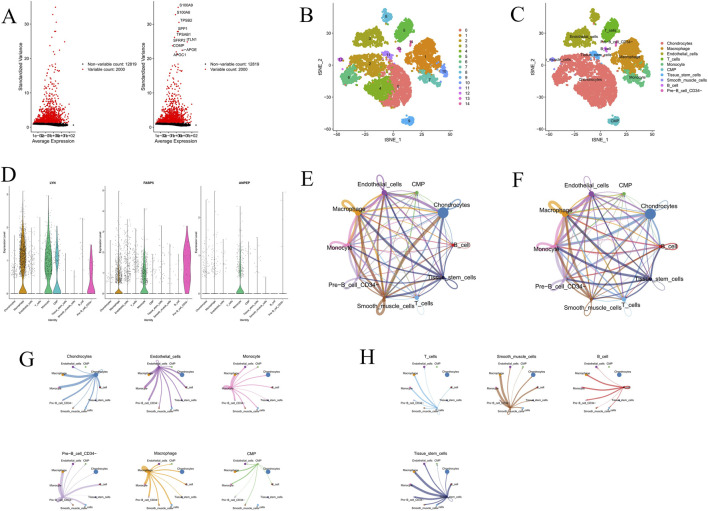
Single-cell RNA sequencing analysis. **(A)** Identification of highly variable genes. **(B,C)** t-SNE plots showing cell clustering and annotated cell types. **(D)** Expression distribution of hub genes *(LYN*, *FABP5*, and *ANPEP*) across different cell types. **(E,F)** Cell–cell communication networks among various immune and stromal cells. **(G,H)** Detailed cell–cell communication interactions for selected cell types.

The immunological microenvironment in AS was characterized by means of bulk sequencing analysis as well as the single-cell RNA sequencing dataset GSE155512. We selected 2,000 highly variable genes for subsequent analysis (Figure 10A). The immune cells were grouped into 15 different clusters (Figure 10B) and classified into nine cell types using the “SingleR” package. These cell types included Chondrocytes, Macrophage, Endothelial_cells, T cells, monocyte, CMP, Tissue_stem_cells, Smooth_muscle_cells, B cells, and Pre-B_cell_CD34- (Figure 10C, Supplementary Material 4). Figure 10D shows in which cell clusters the core genes *LYN*, *FABP5*, and *ANPEP* are enriched. We used the “CellChat” R software to analyze cell-cell interactions in order to interpret variations in intercellular crosstalk within the AS group. Interactions between different cell types were inferred based on the relative expression levels of ligands and receptors (Figures 10E–H). The results revealed that chondrocytes and endothelial cells exhibited particularly close interactions with other cell populations.

### 3.8 Drug prediction results

**FIGURE 11:**
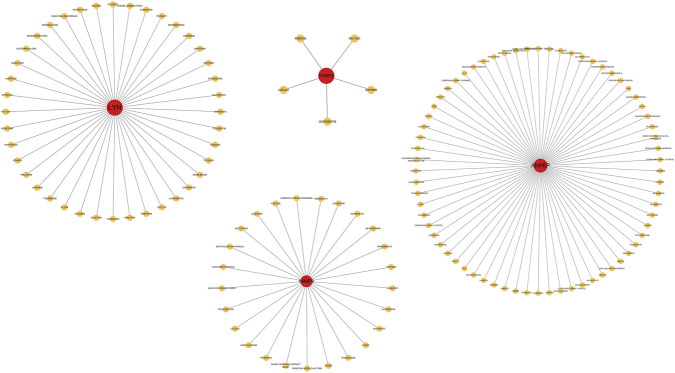
Drug–gene interaction networks of hub genes. The networks illustrate potential drug candidates targeting *LYN*, *FABP5*, *MMP9*, and *ANPEP*, with hub genes shown in red and corresponding drugs in yellow.

To find possible therapeutic drugs that target the identified core genes, we performed queries through the DGIdb database. The results indicated that 40 compounds were associated with *LYN*, 5 with *FABP5*, 25 with *MMP9*, and 68 with *ANPEP*. The corresponding TSV files were retrieved and subsequently imported into Cytoscape for visual representation of the drug–gene interactions, as illustrated in Figure 11.

### 3.9 Prediction of core gene-related TFs and transcriptomic sequencing validation

**FIGURE 12:**
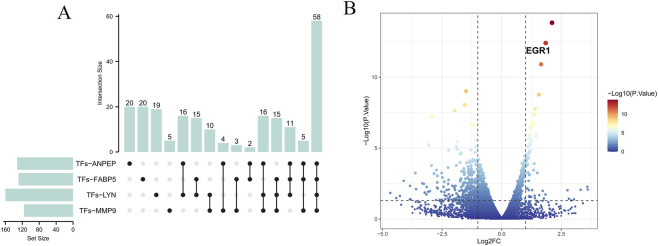
TF analysis of hub genes. **(A)** UpSet plot showing the overlap of predicted TFs regulating *ANPEP*, *FABP5*, *LYN*, and *MMP9*. **(B)** Volcano plot of DEGs highlighting *EGR1* as a key TF.

To find possible therapeutic drugs that target the identified core genes, we conducted queries through the DGIdb database. An upset plot was generated to visualize the overlapping TFs among the four core genes (Figure 12A), resulting in the identification of 58 candidate TFs. Subsequently, transcriptomic sequencing of HUVECs from six samples identified 324 DEGs, among which significantly upregulated or downregulated TF families were determined (Figure 12B). By integrating the 58 overlapping TFs with the transcriptomic sequencing results, we further identified *EGR1* as a central TF (absolute LogFC >1.5, p < 0.0001). In light of these results, we suggest that *EGR1* may control the expression of core genes including *LYN*, *FABP5*, *MMP9*, and *ANPEP*, which would modify MR and affect how AS develops.

### 3.10 Validation of core genes and key TF expression in animal experiments

**FIGURE 13:**
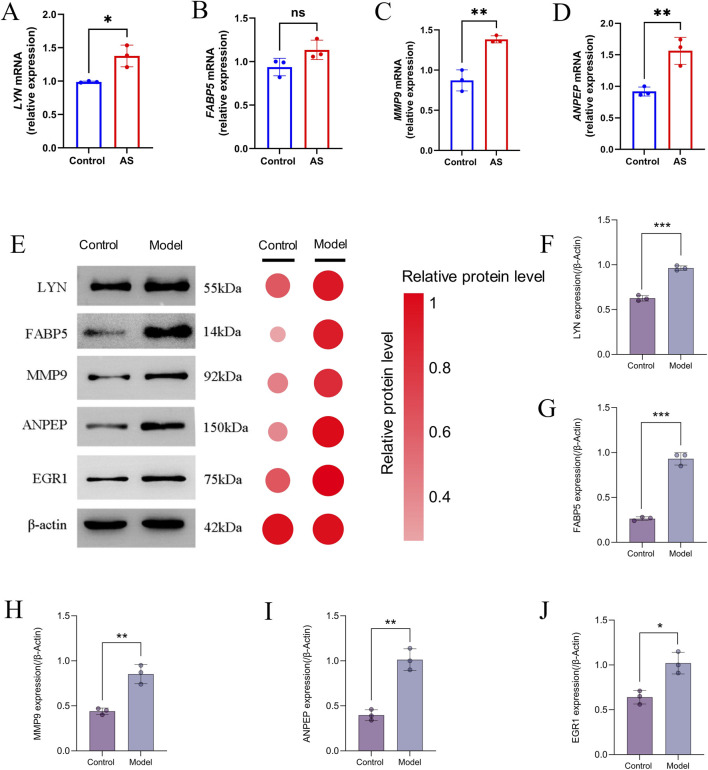
Validation of hub gene expression in the AS model. **(A–D)** Relative mRNA expression levels of *Lyn*, *Fabp5*, *Mmp9*, and *Anpep* in control and AS groups. **(E)** Representative Western blot bands and corresponding semi-quantitative analysis of Lyn, Fabp5, Mmp9, Anpep, and Egr1. **(F–J)** Relative protein expression levels of Lyn, Fabp5, Mmp9, Anpep, and Egr1 normalised to β-actin. Significance levels are indicated as *P < 0.05; **P < 0.01; ***P < 0.001.

We used RT-qPCR and Western blot to compare the expression levels of the core genes *Lyn*, *Fabp5*, *Mmp9*, and *Anpep* in the animal model group with the control group in order to further evaluate the validity of the bioinformatic screening results. Consistent with the patterns seen in the earlier bioinformatic analyses, the results suggested notable changes in these genes' expression in the model group (Figures 13A–I), indicating that they may play important roles in the etiology and development of the disease. Meanwhile, we also assessed the protein expression of the key TF Egr1, which likewise exhibited significantly differential expression in the model group (Figure 13J). These experimental results support the reliability of the identified core genes and TF, and further indicate their important roles in the disease-related molecular mechanisms.

## 4 Discussion

AS, as the primary pathological basis of cardiovascular diseases, has become a major driver of global mortality and disease burden. Despite continuous advancements in the prevention and treatment of cardiovascular diseases, patients with AS still face significant clinical challenges, particularly the difficulty in early diagnosis, due to the current lack of highly sensitive and specific biomarkers. MR is a critical biological process through which cells alter their metabolic patterns in response to environmental stresses to meet energy and biosynthetic demands. In recent years, disease-associated metabolic abnormalities have become a major research focus, with the metabolic features of AS attracting particular attention. Studies have revealed a correlation between the metabolic state of plaques and their clinical risk. In 2002, Rudd et al. reported that symptomatic unstable plaques exhibited significantly higher uptake of [^18^F]-fluorodeoxyglucose compared to asymptomatic plaques, while healthy carotid tissue showed no such uptake (Rudd et al., 2002). This finding was later expanded by Tomas et al., who demonstrated that highly vulnerable plaques display a characteristic metabolic profile: compared to low-risk plaques, they show reduced fatty acid oxidation (FAO) but significantly enhanced glycolysis and amino acid metabolism. Interestingly, these plaques showed much higher levels of mRNA expression for genes involved in the pentose phosphate system and glycolysis. Significantly higher levels of mRNA expression for genes linked to the pentose phosphate pathway and glycolysis were seen in these plaques. This MR signature was significantly associated with a pro-inflammatory state and poorer clinical outcomes, including an increased incidence of cardiovascular events during a 7-year follow-up (Tomas et al., 2018). These pieces of evidence suggest that systematically decoding the regulatory network of MR holds promise for providing new targets for disease diagnosis and treatment. Based on the aforementioned background, this study developed a multi-omics integration framework centered on MR, aiming to systematically identify key regulatory genes and their clinical translational potential in AS. We identified a core gene signature composed of four genes (*LYN*, *FABP5*, *MMP9*, *ANPEP*). This signature was consistently upregulated in AS tissues and showed excellent diagnostic performance in independent validation cohorts. Further analysis revealed that the transcription factor *EGR1* may serve as a common upstream regulatory node coordinating the expression of these core genes. Single-cell transcriptomic and immune infiltration analyses indicated that the expression of these genes was significantly correlated with the enrichment of pro-inflammatory immune cells (e.g., M0 macrophages), suggesting their potential role in shaping the AS immune microenvironment. Finally, experimental validation in an *Apoe* ^*−/−*^ mouse model revealed the significant upregulation of these core genes and Egr1 in AS lesions.

The non-receptor tyrosine kinase encoded by *LYN*, a member of the Src family of kinases, has several regulatory functions in the development of AS. First, by stimulating macrophage scavenger receptors (including CD36 and SR-A), *LYN* encourages the uptake of oxLDL, resulting in intracellular lipid buildup and the generation of foam cells, which is a crucial stage in the development of atherosclerotic plaques (Rahaman et al., 2006). Additionally, *LYN* contributes to fibrillary amyloid-triggered CD36-dependent signaling cascades that increase ROS and tumor necrosis factor-alpha (TNF-α) production, aggravating macrophage inflammatory responses and promoting AS development (Medeiros et al., 2004). *LYN* can increase the production of monocyte chemoattractant protein-1 (MCP-1), which promotes monocyte recruitment and exacerbates vascular inflammation. It also plays a significant role in lipid metabolism (Miki et al., 2001). YOU et al. found that inhibiting *LYN* and its downstream factor Akt increased free fatty acid levels and enhanced mitochondrial fatty acid oxidation, driving macrophages toward an anti-inflammatory phenotype (You et al., 2020). Notably, the role of *LYN* is cell type- and time-dependent; regulated by the SNX10–Lyn–TFEB axis, it can also influence macrophage energy metabolism and phenotypic switching. These studies indicate that *LYN* not only promotes AS progression through inflammatory and lipid metabolic pathways but may also modulate macrophage polarization via the regulation of energy metabolism.

Intracellular lipid chaperones known as fatty acid-binding proteins (FABPs) are extensively expressed in macrophages and adipocytes and are essential for controlling inflammation and energy metabolism (Furuhashi and Hotamisligil, 2008; Furuhashi et al., 2011). *FABP5* is one of those that has attracted a lot of interest because of its crucial roles in AS. Mounting evidence indicates that *FABP5* is not only a potential biomarker for AS but also a promising therapeutic target (Yeung et al., 2008; Bagheri et al., 2010; Hong et al., 2011). Mechanistic studies have demonstrated that loss of *FABP5* alleviates inflammation and modulates monocyte recruitment by activating PPARγ activity in macrophages, thereby effectively attenuating the progression of AS (Babaev et al., 2011). Moreover, *FABP5* levels are closely associated with macrophage cholesterol efflux capacity (CEC), which in turn is inversely correlated with residual risk of atherosclerotic cardiovascular disease (ASCVD). Studies have shown that reduced *FABP5* expression significantly enhances CEC, offering a novel interventional strategy for ASCVD prevention (Furuhashi et al., 2017).

*MMP9* is a key member of the zinc-dependent endopeptidase family and participates in tissue remodeling through the degradation of the extracellular matrix (Nagase et al., 2006). According to clinical research, patients with plaque rupture have noticeably higher plasma MMP9 levels than healthy people (Kosowski et al., 2022). During the development and progression of AS, *MMP9* contributes to pathological changes through multiple mechanisms: (a) regulation of lipid metabolism; (b) exacerbation of vascular inflammation; (c) induction of endothelial dysfunction; (d) promotion of smooth muscle cell proliferation and migration; and (e) acceleration of plaque calcification and destabilization (Brown et al., 2017; Myasoedova et al., 2018; Wang and Khalil, 2018). Existing pharmacological studies further underscore the therapeutic relevance of MMP9. Statins can effectively inhibit collagen degradation in advanced atherosclerotic plaques by downregulating *MMP9* expression (Stasinopoulou et al., 2019). Meanwhile, metformin directly binds to MMP9 and facilitates its degradation, thereby significantly improving plaque stability (Chen X. et al., 2023). These results support the potential of MMP9 as a therapeutic target and suggest its involvement in AS.

*ANPEP* (CD13), a widely expressed ectoenzyme, regulates various biological processes—including peptide activity modulation, phagocytosis, and cholesterol metabolism—by cleaving N-terminal amino acids (Mina-Osorio, 2008). In AS, *ANPEP* exhibits complex and likely cell type-specific regulatory roles. On the one hand, research indicates that *ANPEP* regulates the absorption of cholesterol and could be a target of the medication bexarotene. By downregulating the expression of *Anpep* and *Npc1l1*, bexarotene significantly decreased intestinal cholesterol uptake and enhanced plasma cholesterol levels in an *Apoe*2-KI animal model fed a high-fat diet, thereby slowing the progression of AS (Lalloyer et al., 2006). On the other hand, some evidence indicates that deficiency of *ANPEP* may exacerbate AS. Devarakonda et al. reported that *Anpep*-deficient cells exhibit heightened sensitivity to oxidative stress inducers such as oxLDL, leading to aberrant increases in TFs levels and enhanced cell death, suggesting a protective role of *Anpep* against AS via oxidative stress resistance (Devarakonda et al., 2019). This apparent contradiction may reflect spatiotemporal specificity of *ANPEP* function, implying divergent roles across different cell types or disease stages.

Early Growth Response 1, or *EGR1*, is a zinc-finger TF that is quickly activated in response to a variety of extracellular stimuli, such as mechanical pressures, cellular stress, growth hormones, and inflammatory cytokines. In the development and course of AS, *EGR1* is a crucial regulator. Research has shown that *EGR1* is markedly elevated in both human atherosclerotic lesions and *Ldlr* ^*−/−*^ animal models (McCaffrey et al., 2000). Furthermore, its expression level within plaques shows a strong positive correlation with those of its downstream target genes—such as TGF-β1, TNF-α, ICAM-1, and M-CSF—which supports a potential role for *EGR1* in transcriptional regulation during AS pathogenesis (McCaffrey et al., 2000).

The immune microenvironment of AS is a dynamically evolving inflammatory network, wherein the function and fate decisions of immune cells are tightly coupled with their metabolic states. Recent advances in single-cell and spatial transcriptomics have revealed the substantial heterogeneity of this network in terms of cell types, states, and spatial distributions. For example, studies have identified multiple macrophage subpopulations in human carotid plaques, including pro-inflammatory (e.g., IL1B or C1Q), efferocytotic, proliferative, and SMC-like subsets, alongside highly heterogeneous populations of T cells, SMCs, and fibroblasts (Bashore et al., 2024). A more recent study further employed single-cell RNA sequencing combined with spatial transcriptomics to systematically map senescent vascular cells in a mouse AS model. This work identified distinct senescent subpopulations in vascular SMCs, fibroblasts, and T cells, and found that these subpopulations were enriched in the plaque “cap” and “core” regions (Mazan-Mamczarz et al., 2025). This suggests that senescence, as a special cellular state, constitutes a significant dimension of cellular heterogeneity within the atherosclerotic microenvironment. Classic studies have shown that the metabolic state of immune cells is a determining factor for their phenotype and function. Pro-inflammatory M1 macrophages and effector T cells rely on glycolysis for rapid energy production, whereas regulatory or reparative M2 macrophages and regulatory T cells predominantly utilize oxidative phosphorylation and fatty acid oxidation (Liu et al., 2021). This MR is not only a consequence of cell activation but also serves as the engine driving their specific functions. Notably, single-cell analyses have further unveiled specific associations between this metabolic state and cellular subpopulations. For example, macrophages in symptomatic plaques exhibit higher senescence and glycolytic signatures (Bashore et al., 2024), while intraplaque T cells display a highly activated and dysfunctional state (Horstmann et al., 2024). Similarly, recent research has found that senescent vascular SMCs and fibroblasts highly express genes related to extracellular matrix remodeling, whereas senescent T cells are enriched in inflammatory pathways, indicating that senescent subpopulations of different cell types possess distinct functional and metabolic preferences (Mazan-Mamczarz et al., 2025). In this study, the core genes we identified (*LYN*, *FABP5*, *MMP9*, *ANPEP*) may deeply participate in shaping this imbalanced microenvironment by regulating the metabolic states of immune cells. Specifically, *LYN* and *FABP5*, as key nodes in lipid metabolism and inflammatory signaling, may directly regulate macrophage polarization toward a pro-inflammatory phenotype by influencing their lipid metabolic flux and energy utilization patterns. This could potentially explain why specific macrophage subpopulations (such as those associated with lipid metabolism) correlate with disease severity in single-cell atlases. The expression of *MMP9* itself is driven by upstream pro-inflammatory metabolic pathways (e.g., HIF-1α signaling), and its mediation of extracellular matrix degradation can alter the mechanical and biochemical cues of the microenvironment, thereby affecting the metabolic adaptation and migratory behavior of immune cells (Yang et al., 2019). As an ectoenzyme, *ANPEP* may indirectly regulate the recruitment of immune cells and their surrounding metabolic stress environment by modifying local peptide signals (such as the chemokines CXCL8 and CCL2).

The findings of this study provide a novel perspective on understanding MR in AS, while the identified core biomarkers (*LYN*, *FABP5*, *MMP9*, *ANPEP*) also suggest potential for clinical translation. These genes not only exhibit high diagnostic performance but are also closely associated with the core pathological features of AS, including immune-inflammatory responses, extracellular matrix remodeling, and dysregulated lipid metabolism. Accordingly, they may play multiple roles in the comprehensive management of AS: in the early stage, these markers hold promise as non-invasive or minimally invasive detection indicators, aiding in the identification of pre-clinical or early-stage lesions, particularly enabling more precise risk stratification in populations with traditional risk factors (e.g., hyperlipidemia, diabetes); in disease progression assessment, the multi-gene model constructed in this study can provide molecular evidence for patient risk stratification, helping to distinguish between stable and progressive plaques, thereby guiding the adjustment of individualized treatment intensity; in plaque stability evaluation, the expression levels of core genes (especially MMP9) are significantly correlated with a pro-inflammatory immune microenvironment, suggesting their potential as supplementary biological markers for assessing plaque vulnerability and predicting the risk of acute cardiovascular events. Furthermore, these core genes and their regulatory pathways lay a molecular foundation for developing novel targeted therapies. Predictions based on the DGIdb database indicate that known inhibitors exist for several core genes, pointing the way for drug repurposing or the development of new agents. Of particular importance, the upstream key transcription factor *EGR1* identified in this study, which acts as a “master regulatory switch” coordinating the expression of multiple core genes, provides a highly promising high-level intervention target for developing novel therapies aimed at reprogramming the plaque metabolic-immune microenvironment, with the potential to achieve deeper modulation of disease progression.

Although this study has revealed the critical roles of the core genes (*LYN*, *FABP5*, *MMP9*, and *ANPEP*) and the transcription factor *EGR1* in MR during AS through multi-omics analysis and experimental validation, several limitations should be acknowledged. First, the primary mechanistic validation in this study relied on the *Apoe* ^*−/−*^ mouse model. While this model is a standard tool for AS research, inherent species differences exist between mice and humans in the pathophysiology of AS (e.g., plaque composition, immune responses, and metabolic regulatory networks). Therefore, the universality and translational value of the core gene regulatory network identified here, particularly the upstream dominant role of *EGR1*, require further confirmation using clinical samples or models that more closely resemble human pathology (e.g., organoids or humanized models). Second, research based on high-dimensional omics data and complex bioinformatics pipelines inherently carries a risk of false-positive findings. Although we employed multiple dataset integration, cross-validation with various machine learning algorithms, and independent animal experimental validation to maximize the robustness of our screening results, the association between the selected biomarkers and AS remains observational and predictive. The precise causal roles of these genes in disease pathogenesis and progression, as well as the specific molecular mechanisms by which *EGR1* transcriptionally regulates them, ultimately require confirmation through orthogonal experimental strategies such as functional gain-/loss-of-function experiments (e.g., CRISPR-Cas9 gene editing) coupled with phenotypic rescue studies. Future research should combine multi-center clinical cohorts to validate the diagnostic potential of these biomarkers and employ cell type-specific gene-editing technologies to dissect their functions in depth. Simultaneously, targeted intervention strategies based on the core genes should be explored, integrating spatial transcriptomics, metabolomics, and epigenomics data to comprehensively unravel the regulatory network of MR in AS and provide novel strategies for precision therapy.

## 5 Conclusion

This study established a multi-omics and machine learning integrated analytical framework centered on MR to systematically identify and validate four core genes (*LYN*, *FABP5*, *MMP9*, and *ANPEP*) that play critical roles in AS. Our analysis suggested that the transcription factor *EGR1* could be an upstream regulator of these genes. Furthermore, the altered expression of these core genes was validated in an animal model. By integrating single-cell sequencing and immune infiltration analysis, this study elucidated the close association between these core genes and the remodeling of a pro-inflammatory immune microenvironment. In summary, these findings not only deepen the understanding of the pathogenesis of AS from the novel perspective of “metabolism-immune” interactions but, more importantly, the identified gene signature and its regulatory network provide crucial molecular targets and a theoretical foundation for developing novel biomarkers applicable to the full-cycle management of AS, as well as for devising precision therapeutic strategies targeting MR.

## Data Availability

The datasets presented in this study can be found in online repositories. The publicly available transcriptomic datasets analysed in this work were obtained from the GEO database under the accession numbers GSE100927, GSE28829, GSE43292 and GSE155512. In addition, the RNA-sequencing data generated in this study have been deposited in the Figshare repository and are accessible under the DOI 10.6084/m9.figshare.30736481.
