# Supplementary material for: Identification of core genes and transcription factors related to metabolic reprogramming in atherosclerosis: a multi-omics analysis and experimental validation approach
Source: Front Mol Biosci. 2026 Feb 25;13:1756851. doi: 10.3389/fmolb.2026.1756851 (PMC12975476; doi:10.3389/fmolb.2026.1756851)
Supplement: Supplementary file 2 [file Supplementaryfile3.docx]

| Primer Name | Primer Sequence(5'-3') | Fragment Length (bp) | Annealing Temperature (°C) |
| --- | --- | --- | --- |
| M-LYN-S | TTAGAAGTCTGGACAATGGTGGCT | 148 | 60 |
| M-LYN-A | GGCTTCTGAGGTTTGGGACTGA |  | 60 |
|  |  |  |  |
| M-Fabp5-S | GCCAGTCTTAAGGATCTCGAAGG | 170 | 60 |
| M-Fabp5-A | CTCTCGGTTTTGACCGTGATGT |  | 60 |
|  |  |  |  |
| M-MMP9(4)-S | GCTGGCAGAGGCATACTTGTAC | 162 | 60 |
| M-MMP9(4)-A | GGTGTTCGAATGGCCTTTAGTG |  | 60 |
|  |  |  |  |
| M-Anpep-S | GTGGTGGCTACAACGCAGAT | 150 | 60 |
| M-Anpep-A | CTTGGACTCTTTGGGAAGCATA |  | 60 |
