# Supplementary figures and images for: Identification of core genes and transcription factors related to metabolic reprogramming in atherosclerosis: a multi-omics analysis and experimental validation approach
Source: Front Mol Biosci. 2026 Feb 25;13:1756851. doi: 10.3389/fmolb.2026.1756851 (PMC12975476; doi:10.3389/fmolb.2026.1756851)

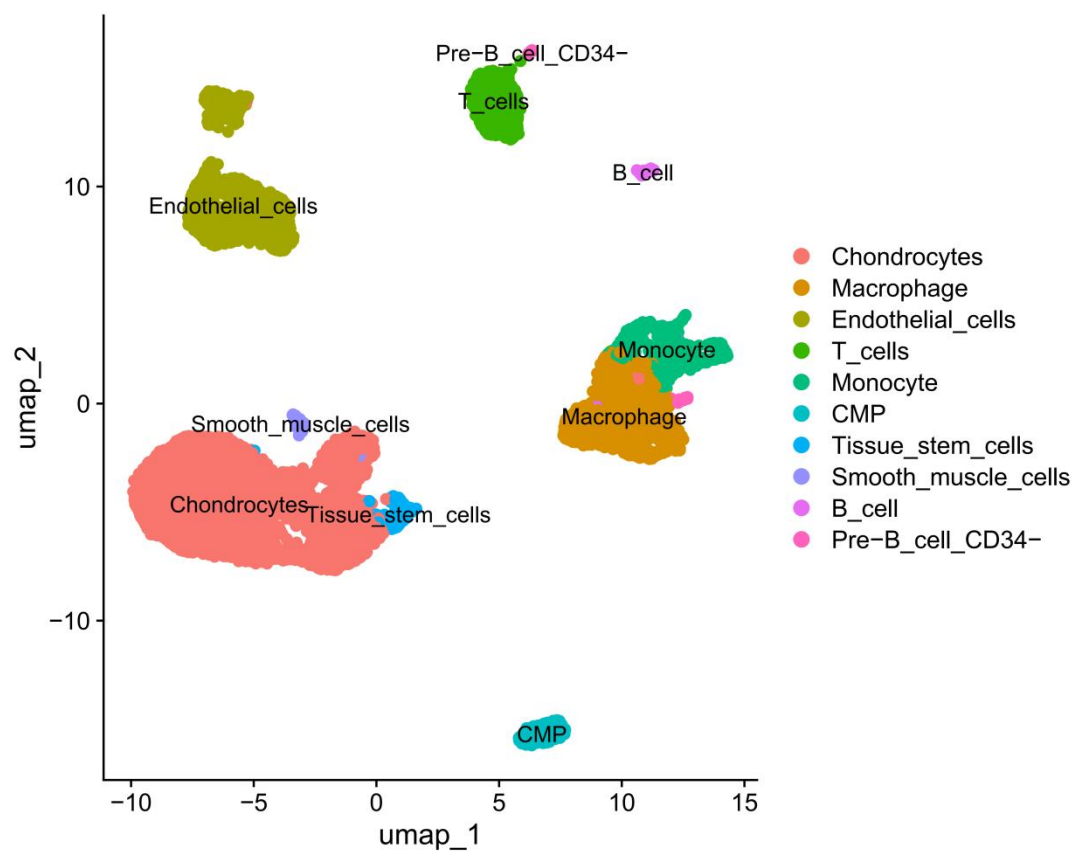

#### Supplementary File 4

U-map plots showing cell clustering and annotated cell types.

Supplement: Supplementary file 4 [file Supplementaryfile4.pdf]
